# Recent Advances in Phospholipids from Colostrum, Milk and Dairy By-Products

**DOI:** 10.3390/ijms18010173

**Published:** 2017-01-17

**Authors:** Vito Verardo, Ana Maria Gómez-Caravaca, David Arráez-Román, Kasper Hettinga

**Affiliations:** 1 Department of Chemistry and Physics (Analytical Chemistry Area), Research Centre for Agricultural and Food Biotechnology (BITAL), Agrifood Campus of International Excellence, ceiA3, University of Almería, Carretera de Sacramento s/n, 04120 Almería, Spain; vito.verardo@unibo.it; 2 Department of Analytical Chemistry, University of Granada, c/Fuentenueva s/n, 18071 Granada, Spain; anagomez@ugr.es; 3 Research and Development of Functional Food Centre (CIDAF), Health Science Technological Park (PTS) Granada, Avda. del Conocimiento s/n, EdificioBioregión, 18007 Granada, Spain; 4 Dairy Science and Technology, Food Quality and Design Group, Wageningen University, Bornse Weilanden 9, 6708 WG Wageningen, The Netherlands; kasper.hettinga@wur.nl

**Keywords:** colostrum, milk, phospholipids, dairy by-products, healthy effects

## Abstract

Milk is one of the most important foods for mammals, because it is the first form of feed providing energy, nutrients and immunological factors. In the last few years, milk lipids have attracted the attention of researchers due to the presence of several bioactive components in the lipid fraction. The lipid fraction of milk and dairy products contains several components of nutritional significance, such as ω-3 and ω-6 polyunsaturated fatty acids, CLA, short chain fatty acids, gangliosides and phospholipids. Prospective cohort evidence has shown that phospholipids play an important role in the human diet and reinforce the possible relationship between their consumption and prevention of several chronic diseases. Because of these potential benefits of phospholipids in the human diet, this review is focused on the recent advances in phospholipids from colostrum, milk and dairy by-products. Phospholipid composition, its main determination methods and the health activities of these compounds will be addressed.

## 1. Introduction 

Milk chemical composition varies depending on, e.g., the mammal species, genetics, environmental factors, lactation stages, feed composition and nutritional status. This variation is mainly quantitative, because the major components in all milks are water, sugars (particularly lactose and oligosaccharides), proteins, lipids, minerals and vitamins [1].

Different lipid classes compose the total milk fat fraction; however, as noticed in other lipid matrices, triglycerides represent the most abundant class, being 97%–98% of total milk fat [2].

Milk lipids are organized in a macrostructure composed of globules made up of triglycerides with different melting points as its core, enveloped by three membrane layers called the milk fat globule membrane (MFGM) [3,4]. The membrane is comprised of many different bioactive compounds, such as lactoferrin, IgG, sialic acid and a range of unique polar lipids. The most important polar lipids contained in the MFGM are the phospholipids [2,5,6].

Milk phospholipids could be divided into two major classes called glycerophospholipids and sphingolipids [2,5], together representing about 1% of the total milk lipid fraction [2].

**Figure 1:**
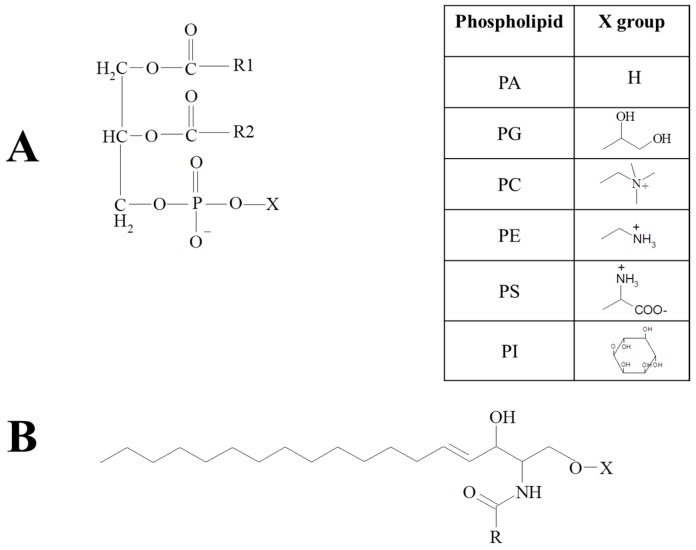
Structure of the main glycerophospholipids (**A**) and sphingolipids (**B**).

As shown in Figure 1A, glycerophospholipids consist of a glycerol backbone on which an *O*-acyl, *O*-alkyl or *O*-alk-1′-enyl residue is bound at the sn-1 position and an *O*-acyl residue at the sn-2 position of the glycerol moiety. A phosphate residue with different combinations of polar heads at the sn-3 position differentiates the different phospholipids [5]. The lysoglycerophospholipids can be obtained when specific enzymes partially hydrolyze the glycerophospholipids, removing one fatty acid group.

Sphingolipids (Figure 1B) are formed of a sphingoid base, a long-chain aliphatic amine containing two or three hydroxyl groups, a long-chain fatty acid linked to the amide and a polar head group [2,5].

This review summarizes the information that became available in the last sixyears on phospholipids contained in colostrum, milk and dairy by-products, as well as their health effects. For that, phospholipid composition, analytical approaches for their determination and in vitro and in vivo biological activity have been addressed.

## 2. Colostrum, Milk and Dairy By-Product Phospholipid Composition

As mentioned above, milk phospholipids are mainly localized in the milk fat globule membrane, which is composed of 60%–70% phospholipids. However, technological processes (mainly homogenization) disrupt the fat globule, thereby destroying the membrane. Therefore, after dairy processing, the phospholipids are no longer only associated with the fat globules [6].

Table 1 shows the presence of individual phospholipids and total phospholipid concentration as determined in colostrum, milk and dairy by-products of several mammalian species.

### 2.1. Phospholipid Content and Composition in Human Colostrum and Milk

Literature information on phospholipids content in human colostrum is scarce. However, some important information about the phospholipids evolution in colostrum and human milk have been reported. Zou and co-workers [7] compared the phospholipid content in colostrum, transitional and mature milk of Danish mothers. Significant differences in phospholipid content were found at different stages of lactation. PC and SM were the main phospholipids in human colostrum and milk. Sphingomyelin did not show significant changes over lactation, whereas the authors observed that PC content in colostrum and transitional milk was significantly lower than in mature milk. This trend was attributed to the changes of mean diameters of human MFG from colostrum to mature milk; it was shown that MFG diameter decreased from colostrum to milk, according to Lopez et al. [3], and the total concentration of polar lipids in milk is inversely correlated with MFG diameter. PI, PS and PE increased from colostrum to milk [3]. Regarding fatty acids that form phospholipids, C16:0 was the most abundant fatty acid of the colostrum and milk phospholipids, followed by C18:1 ω-9, C18:2 ω-6 and C18:0. Generally, saturated fatty acids constituted more than 60% of total phospholipid fatty acids, which is similar to milk triglycerides. C16:0 content decreased from colostrum to milk, whereas the opposite trend was shown for C18:0. ω-3PUFAs were higher in milk than in colostrum [3]. Several authors [7,10] also described that saturated fatty acids were the major constituents of phospholipids; however, Zou et al. [7] reported C16:0 as the most abundant fatty acid in human colostrum, transitional milk and mature milk phospholipids, which contradicts Benoit et al. [10], who found stearic acid (C18:0) as the first PL fatty acid followed by C18:2 ω-6, C16:0 and C18:1 ω-9, respectively. These discordances could be due to a different sampling; in fact, Zou et al. [7] analyzed human colostrum, transitional milk and mature milk samples after 16 days of lactation; instead, Benoit et al. [10] obtained their samples from volunteer mothers of term infants after 3–6 months of exclusive breastfeeding, thus reporting data much later in lactation. As reported by Zou et al. [7], C16:0 and C18:0 fatty acids showed an opposite trend from colostrum to milk; briefly, higher amounts of stearic acid were found in human milk compared to colostrum. Moreover, the same authors reported ω-6 PUFA to increase during lactation [7].

Recently, Claumarchirant et al. [8] evaluated the phospholipid contents in colostrum, transition and mature milk of mothers from two different Spanish geographic areas. Statistical differences were noticed among the lactation stages, and according to Zou et al. [7], transitional milk had the highest concentration of phospholipids. The authors also analyzed the MFG diameters, and they found them smaller in transitional milk, followed by mature milk and colostrum, respectively. This correlation between MFG diameter and PL content is as expected, as it is known [3] that polar lipid content is negatively correlated with the diameter of MFG, because more PL per unit fat would be needed to cover smaller globules. The main phospholipid class found in these milks was SM, followed by PE, PC, PS and PI. Moreover, the authors observed that the content of minor phospholipids (PI, PS and PC) was higher in the coastal zone than in the central zone, whereas they did not find significant changes in PE and SM content. Although the reason is unknown, dietary differences may underlie these compositional differences.

Other authors [7,8,10,11,12,14] also studied the phospholipid content in human milk. All of them agreed about the fact that SM is the most abundant phospholipid in human milk, ranging from 29.7%–45.5% of total phospholipids. However, different data have been reported about the second and third human milk phospholipid. PC was described as the second PL by some authors [7,11,12], whereas PE was the second one determined by several other authors [8,10,14]. Different PL compositions were found by Russo et al. [13] that described PS and SM as the first and second PL, respectively. The different studies identified PC [8,10,14], PE [12,13] or PS [7,11] as the third phospholipid in human milk. These discrepancies could be mainly due to the different characteristics of the samples (geographical origin, diet, cultural traditions, lactation stage).Several authors also described the presence of the lyso form of phospholipids [5,12,13]; their content is lower than 12% of total phospholipids.

### 2.2. Phospholipid Content and Composition in Bovine Colostrum and Milk

As expected, the literature about bovine milk and its derivatives is more abundant than the literature about human milk. Contarini et al. [16] and Zuo et al. [17] demonstrated that total phospholipid content in bovine colostrum is in the same order of magnitude as human colostrum. As noticed by Contarini et al. [16], total phospholipid content decreased after 24 h postpartum and did not change from 48 h to fifty days of lactation. SM was the main PL in bovine colostrum, and its content decreased during lactation. After 24 h, PC and PE were the second and third PL, respectively; but their order inverted after 96 h post-partum. No quantitative change was noticed for PC; contrarily, PE content increased during lactation. Zuo et al. [17] also analyzed bovine colostrum and milk phospholipids; PE followed by SM and PC represented about 78% of total phospholipids. In their study, contrary to Contarini et al. [16], total PL increased from colostrum to milk; the same trend was reported for PE, PI and PS. No statistical differences were found for PC, but SM decreased from colostrum to milk. This decrease is caused by the differently-sized fat globules [3,17]. Kiełbowicz et al. [24] also analyzed different cow milk samples, and they determined PC and PE as the principal phospholipids.

Lopez and co-workers [3] analyzed phospholipids in whole bovine milk and after membrane separation of small and large MFG. PC, SM and PE were the main phospholipids in all samples. In addition, they noticed that total phospholipid content in small MFG was 1.4-fold more than in whole milks and 3.5-fold more than in large MFG, which is to be expected due to the larger surface-to-volume ratio of small MFG. However, small MFG presented lower relative proportions of PC and SM. The most probably hypothesis is that the secretion of small MFG can induce important changes in the local curvature of the bilayer membrane that produce a desorption and then loss of some membrane materials, such as PC and SM. Moreover, due to the different localization of the polar lipids in MFGM (PE, PS and PI are mostly located in the inner surface, whereas PC and SM are located in the outer bilayer of the MFGM), it is possible that PC and SM may be excluded from the MFG membrane of smaller globules, due to the greater curvature of their surface [3].

Gallier et al. [18,20,21] determined several phospholipids in bovine milk; PC, PE and SM were the first, second and third phospholipid, respectively, in their study. Moreover, they found that total fatty acid distribution in phospholipids was in the following order: polyunsaturated > saturated > monounsaturated, which is different from human milk, for which saturated fatty acids were reported to be the most abundant. SM was the most saturated phospholipid (containing C16:0, C22:0 and C24:0 as the main fatty acids), and PE showed the highest polyunsaturation ratio. Zou et al. [25] also found PE, PC and SM as the main cow milk phospholipids; nevertheless, they reported that saturated fatty acids constituted about 57% of total phospholipid fatty acids, followed by monounsaturated and polyunsaturated fatty acids.

Mesilati-Stahy et al. [23] showed similar results in terms of phospholipid composition, but only when large-sized MFGs were analyzed; however, they demonstrated that this order changed when small-sized MFGs were considered. They showed that SM was the most abundant phospholipid in the MFGs with a diameter smaller than 2 μm, followed by PC, PI and PE. 

As can be seen, several factors could influence the phospholipids and their fatty acid composition. Argov-Argaman et al. [30,34] reported that cow diet can influence the MFG diameter; particularly, low concentrate/high-forage diet of cows caused an increase in MFG diameter. Moreover, they found that higher PS concentrations were reached when larger globules were produced. The same authors [32] also demonstrated that PC was not affected by MFG size and lactation stage; on thecontrary, PE was negatively affected when MFG size decreased. 

Mesilati-Stahy [37] examined the effect of glucogenic dietary supplementation and reproductive state on milk phospholipids. They demonstrated that phospholipid composition was affected by the reproductive state; in fact, PE was about 1.5–2-fold higher in the first day compared to eightdays post-estrus. PE concentration in milk was dependent on the interaction between glucogenic dietary supplementation and reproductive state.

Higher amounts of polar lipids (+72%) were noticed by Lopez et al. [31] when cows were fed with a fresh pasture-based diet during spring; this trend was justified by the larger proportion of small fat globules that were secreted in spring milk, which require more PL to cover the additional MFG surface. PC, PE and SM were the most abundant phospholipids, and the seasonal variations of the cow diet did not favor the synthesis of a specific kind of polar lipid. No statistical differences were therefore found in the relative proportions of the phospholipid classes in the samples collected in spring or in other seasons. On the other hand, the fatty acid composition of the phospholipids was significantly affected by the season; spring milk phospholipids contained a lower amount of saturated fatty acids, probably due to the overall lower level of saturated fatty acids in milk during grazing.

An increase of phospholipid content was also reported by Ferreiro and co-workers [38] who compared conventional milk with organic milk. The relative proportions of the phospholipids descended in the order PE > PC > SM > PS > PI. These changes were attributed to the different composition of the diet and different level of physical exercise of cows.

Recently, also the contribution of the genetics of the cow on polar lipid composition was studied. As reported by Argov-Argaman and co-workers [52], milk total polar lipid content, as well as specific lipid constituents in the polar lipid envelope of the MFG are influenced by diacylglycerol acyltransferase 1 (DGAT1) K232A polymorphism. Their results showed that SM, PI and PS were associated with a genotype*fat content interaction, indicating that polar lipid composition is related to genotype, in a fat content-related manner. The results obtained by these researchers encourage the selection of selective breeding in order to obtain naturally PL-enriched milk. The lysophospholipids content of cow milk and its dairy by-products is lower than 2% of total phospholipids [5,12].

Briefly, high variability between bovine colostrum and milk was noticed; different factors such as lactation stage, genetics and feeding are the many variables that influence the MFGM composition and, subsequently, the PL composition and content. This indicates that farm management may be a powerful tool to change both total PL content, as well as PL composition.

### 2.3. Phospholipid Content and Composition in Other Mammalian Milks

Garcia et al. [12] compared the phospholipid composition of human, cow, camel and mare milk. The highest phospholipid content was noticed in camel milk ranging from 257.0–660.3 µg/mL; on the contrary, mare milk reported the lowest content with a median of 77.8 µg/mL. The % of PL compared to total lipid was in the following order: mare > camel > human > cow. SM and PC were the most abundant phospholipids in human and mare milk; PE and SM were, however, the first and second phospholipids, respectively, in cow and camel milk. Russo et al. [13] also compared the phospholipid profile among cow, goat, donkey and human milk. Goat milk showed a phospholipid content comparable with human and pasteurized cow milk, but donkey milk phospholipids were five-fold lower than in the other milks analyzed. Donato et al. [22] confirmed this trend. Russo et al. also studied the phospholipid molecular species composition of the different milks, and they reported that PI C18:0/C18:1, PE C16:0/C18:1, PS C16:0/C18:1 and PC C16:0/C18:1 were the main phospholipids in the respective classes. C16:0 is the principal fatty acid in SM and lysoPC in all milk samples.

Rodríguez-Alcalá and Fontecha [19] determined phospholipids in cow, goat and ewe milk. They reported that phospholipids descended in the following order PE > PC > SM in all three samples. Zou et al. [25] also analyzed the phospholipid profile of human, cow, buffalo, sheep, donkey and camel milk. Buffalo milk showed the lowest phospholipid content; however, no significant differences were found among the other ones. SM in cow, buffalo, donkey, sheep and camel milk fats was significantly lower than that in human milk, whereas PE was the least abundant phospholipid in human milk.

Zancada and co-workers [50] compared the phospholipid fatty acids profile of ovine and caprine milk. Their results showed that SFAs were the major fatty acids in phospholipids of both milks, and goat milk showed the highest percentage of phospholipids (65% vs. 52%). On the contrary, the highest percentage of mono- and poly-unsaturated fatty acids wasfound in ovine milk phospholipids. In both samples, SM contained more than 96% of SFA and PE more than 51% of MUFA. Recently, Argov-Argaman et al. [51] determined phospholipid content and composition in goat milk collected at different lactation stages and fed in two different ways (randomly to grazing in Mediterranean brush land or fed clover hay indoors, in addition to concentrate). They demonstrated that PL composition was significantly different between the two feeding strategies. Briefly, PI and PC were affected by lactation stage and fed.

### 2.4. Phospholipid Content and Composition in Dairy By-Products

Buttermilk is a by-product from butter production, and it is the main dairy by-product that has been analyzed for its phospholipid content, because during butter making, the membrane is separated from the milk fat, and buttermilk may thus be a good source of phospholipids. In fact, Costa and co-workers [41] observed that buttermilk contains about 12% of phospholipids (expressed astotal fat), and PC, SM and PE constitute more than 84% of total phospholipids. Moreover, they achieved concentering the phospholipid fraction to 61% (based on fat) using a combination of membrane filtration and supercritical fluids technologies. Konrad et al. [44] also applied ultrafiltration on whey buttermilk in order to obtain phospholipid-enriched fractions. With this strategy, they obtained a dried fraction with a phospholipid concentration up to 14% (*w*/*w*). A similar approach was performed by Svanborg et al. [46] who determined PC and PE as the main phospholipids in buttermilk and microfiltered buttermilk.

Verardo et al. [43] analyzed the phospholipid content in buttermilk collected in the protected designation of origin Parmigiano Reggiano cheese area. They considered two kinds of samples: buttermilk from butter production by Fritz method and Reggiana cows’ buttermilk obtained by traditional churning. Buttermilks showed a similar composition of individual phospholipids (PE > PC > PS > SM > PI); however, samples from Reggiana cows, and manufactured by the traditional churning process, had the highest phospholipid content. This could be due to the influence of the different diets between Reggiana cows (fed only with forage) and the other cows. These authors also analyzed the phospholipid degree of unsaturation and reported the order to be saturated > monounsaturated > polyunsaturated. A similar phospholipid composition (PC > PE > PS > SM > PI) was found in buttermilk powder [19]. Instead, Gallier et al. [20,21] showed that PC and SM were more than 68% of total phospholipids in powder buttermilk. This discrepancy could be due to different sample characteristics (lactation stage, fed, etc., important factors as discussed before). In addition, they studied the degree of unsaturation of the phospholipids present in buttermilk powder and found that more than 40% were polyunsaturated.

Barry et al. [39] compared the phospholipid content in buttermilk and butter serum; total phospholipids content (expressed on fat) was higher in butter serum than in buttermilk (46.1% vs. 35.3%). Only a few differences were found in phospholipid composition between the two dairy by-products; briefly, PE, PC and SM were the main phospholipids in both products. However, PC was the first phospholipid in buttermilk, and SM was the most abundant one in butter serum. This difference could be due to the different locations of these PL in MFG; in fact, PC is present in the inner layer of MFG and is thus probably released in buttermilk during churning. SM is organized in a rigid domain, which could permit its association with the milk fat during churning and, therefore, its presence in butter serum.

Zhu and Damodaran [48] studied mozzarella cheese whey. The major phospholipids were PC, PE and SM, whereas PS and PI were the minor components. Saturated fatty acids represented 46% of total phospholipid fatty acids, with palmitic, oleic, steric and meristic being the main fatty acids.

Guerra et al. [47] analyzed the phospholipid composition in different cream samples (a by-product of industrial Parmigiano Reggiano cheese-making); cream samples were obtained from raw milk collected after supplementation of the cows’ diet with extruded linseed. These authors noticed as linseed supplementation increased, the CLA and DHA content in cream phospholipids increased, as well.

## 3. Analytical Approaches for Phospholipids Determination in Colostrum, Milk and Dairy By-Products

Current interest on phospholipids in colostrum, milk and dairy by-products hasgreatly increased the developments and applications of different chemical extraction and determination methods. The main used extraction methods and analytical determination methods are summarized in Table 2.

As reported in Table 2, the Folch method was the most widely-used extraction protocol for phospholipid extraction [53]. Only a few authors used alternative protocols, such as the modified Folch method [8,15,28,33,35,36,51], Röse-Gottlieb [39,44] and Bligh and Dyer [26,29,45,46,50] protocols. These protocols have in common that polar organic solvents, such as chloroform, dichloromethane and methanol, are used in order to extract the polar lipid fraction. Alternative methods of extraction were tested by Costa et al. [41], using ether to extract the phospholipids, and by Zhu and co-workers, whoextracted the phospholipid fraction with ethanol [48].

After lipid extraction, it is sometimes necessary to further purify the polar lipid fraction as a second step. This purification step can be necessary when there is a low content of phospholipids (<1%) in the total lipid extract [5]. However, the need forpurification also depends on the combination of phospholipid concentration and the analytical method that will be used to determine the phospholipid fraction. To support this hypothesis, Verardo et al. [43] screened the performance of three SPE stationary phases (C8, silica and zirconia) in order to corroborate the recovery of phospholipids from milk lipids. The silica SPE column showed the best recovery, and it was therefore used before the phospholipid analysis with ammonia solution in the mobile phases. Rombaut and co-workers [54] proposed an HPLC method where they substituted the ammonia solution with trimethylamine buffer (pH 3, 1 M formic acid); this method allowed a successful separation of phospholipids in milk and dairy products without a purification step by SPE [43,54,55]. However, this method without prior purification should not be used when the phospholipids are analyzed by HPLC-MS, in order to avoid the persistent memory effect of triethylamine in mass spectrometers [47].

Usually, normal phase chromatography has been used for phospholipid HPLC separation [5] and silica and diol have been the most used stationary phases. However, aqueous mobile phases alter the affinity between analytes and the stationary phase during the use of the column; because of that, the analyses are not totally reproducible, and the column life is short due to the need to use extreme conditions [56]. In the last few years, the use of hydrophilic interaction chromatography (HILIC) is increasing in popularity [13,22,27,28,35] to avoid this disadvantage of regular HPLC. This HILIC methodology uses polar stationary phases compatible with water-miscible solvents (contrary to regular silica columns); this allows more reproducible analyses and, at the same time, increases the column life. Recently, Walczak et al. [40] proposed a new HILIC approach to separate milk phospholipids using a column with home-made phosphoester chemically bonded stationary phase containing diol, phosphate and octadecyl groups, obtaining good selectivity and less analysis time compared to regular silica columns.

Evaporative light scattering detection (ELSD) is the most widely-used detector to determine phospholipids in food [53]. As reported in Table 2, it is also extensively applied to the analysis of phospholipids in milk and dairy by-products. Despite its low selectivity, low sensitivity and its operation as a destructive detector, it presents several advantages that make it popular for phospholipid analysis. Among the advantages, it is less expensivecompared with other detectors, easy to use, has a wide dynamic linear range and furnishes a similar response factor for molecules with similar structure [53]. One of the main disadvantages of phospholipid determination using ELSD is that standard compounds are needed to identify the analytes. Alternatively, good results were also obtained coupling HPLC with the charge aerosol detector (CAD) [24,39].

However, both ELSD and CAD detectors do not provide any information about molecular species that constitute the phospholipids. Because of that, comprehensive analysis of phospholipids is essential to enhance the knowledge about the phospholipid composition of milk and dairy products. Thus, several methods have been developed using HPLC coupled to mass spectrometry detectors [13,22,28,35,40,42,43,47]. In this way, it is possible to furnish additional information about the molecular species (especially the fatty acids) that are bound to the glycerol backbone. A recent advance has been reported by Dugo and co-workers [27] that proposed a new method based on comprehensive two-dimensional liquid chromatography coupled to mass spectrometry. The authors used a HILIC column in the first dimension and an RP C18 column in the second dimension. With this analytical design, they obtained the separation of several phospholipid molecular species in cow milk. Moreover, they resolved the co-elution of molecular species with the same molecular weight, but different fatty acid composition (i.e., PE C18:0/C18:2 from PE C18:1/C18:1).

Other analytical strategies comprise the direct infusion, after electrospray ionization, of milk or dairy fat in mass spectrometers [9,18,20,21], or a coupled MALDI-mass spectrometry system [26,29,45]. However, the data interpretation in these cases is more difficult, because the different phospholipid classes have not been separated prior to mass spectrometry.

If the mass spectrometry is able to furnish information about the molecular species that are bound to the phospholipid molecules, phospholipid separation is needed in order to quantify the phospholipid fatty acid content. TLC represents a good option to separate the phospholipid fraction from the other lipid classes [7,10,25,43,48,50]. In fact, after milk fat elution, the phospholipid band was located at the origin since it did not migrate with the solvent mixture used; because of that, it is scraped-off, and the phospholipids extracted from this band are transmethylated and analyzed by GC.

Recently, 31P NMR has become a valuable technique for the analysis of phospholipids in dairy foods [57] due to several advantages over MS, such as its non-destructive nature and the fact that it does not require any standard components to identify the PL compounds. This technique is preferred over 1H NMR techniques for its good resolution and the absence of overlap with the signal of neutral lipids [58]. Gallier et al. [59] and Zhu and Damodaran [48] determined PE, PC, PE, PS and SM in bovine milk fat globules and in dairy lecithins from milk whey, respectively, using this technique. Garcia and co-worker [12] screened the phospholipid content in human, cow, camel and mare milk by 31P NMR; their proposed method was reported to have easy sample preparation, high resolution and good sensitivity, permitting the identification and quantification of 12 phospholipid compounds, including some lyso-phospholipids.

A complete different research direction is the study of native MFGM composition and structure. In the last decade, several authors have used confocal laser scanning microscopy to study the phospholipid composition and distribution in MFGM of milk and dairy products. Briefly, the phospholipids in the MFGM were labelled by Rd-DOPE to assess the distribution of these compounds in MFGM. This technique was used to determine the phospholipids in human [7,11,60], bovine [3,17,20,21,59], buffalo colostrum [61], milk and dairy by-products.

Using this technique, the authors were able to determine the composition of MFGM, as well as the lateral organization of their MFGM, according to their size. Zou et al. [17] found heterogeneous domains in the MFGM that could be the result of the lateral segregation of SM at room and physiological temperature. These results improved the knowledge about the lipid composition of the MFGM and its microstructure at different temperatures. This information could be useful for food formulation, because foods are usually stored at much lower temperatures thanthe normal body temperature of dairy animals.

Current advances were also reported using confocal Raman microscopy [59]; Yao et al. [62] detected that the polar head groups of phospholipids showed bands around 860 cm^−1^, and phospholipids unsaturation was represented in bands at 1270 and 1303 cm^−1^. Based on these data, the authors found that colostrum contained higher concentration of phospholipids than milk. They could also discriminate among human, bovine and caprine milk. This analytical approach is very interesting, because confocal Raman microscopy is a non-destructive method and can be applied on-line; however, it needs a previous setup and validation using a large prior sampling to develop the classification models used for correct identification.

To summarize, different analytical approaches can be used to determine the milk and dairy phospholipids; each one furnishes different information that sometimes overlaps and other times is complementary. The use of one technique among others depends on the scope of the research and the instrumental availability.

## 4. Health Benefits Provided by Colostrum, Milk and Dairy By-Product Phospholipids

The consumption of milk and dairy products has been related to the intake of high amounts of saturated fatty acids during the years. However, several authors reported that consumption of dairy products improves the intakes of many nutrients and reduces the risk of several diseases, such as heart disease, hypertension, obesity and type 2 diabetes and cognitive function [63,64,65,66,67,68,69]. In fact, Sébédio and Malpuech-Brugère documented an inverse relation between the consumption of dairy products and the prevalence of metabolic syndrome [70]; moreover, Astrupand et al. [71] noticed that the consumption of low-fat dairy foods does not contribute to improve body composition and metabolic health when compared to regular fat dairy foods. Díaz-López and co-workers [72] reported that a healthy dietary pattern incorporating a high consumption of dairy products, in particular yogurt, may be protective against type 2 diabetes in the elderly at high cardiovascular risk.

Some of these beneficial effects of dairy products are attributed to the phospholipids contained in milk and its derivatives [6,73,74,75]; because of that, a summary of the research studies carried out about the beneficial effects of these compounds is reported below.

Among others, studies conducted by Haramizu and co-workers [76] demonstrated that long-term MFGM (a rich source of phospholipids) intake combined with regular exercise improved endurance capacity; this activity is due to an increased lipid metabolism modulated by SM contained in MFGM. Moreover, the same authors [77] also attributed the improvements of neuromuscular development and IGF-1 signaling in senescence accelerated mice to MFGM phospholipids. Furthermore, other principal activities of milk and dairy phospholipids are noticed as discussed below.

### 4.1. Activity on Neurological and Neurocognitive Diseases

The effects of milk phospholipids on the neurological and cognitive system have widely been described [78,79,80]. Liu and co-workers [81] evaluated the effect of a milk protein concentrate with a minimum of 16% naturally-derived phospholipids. They found that supplemented piglets reported higher learning and memory ability compared with control piglets; moreover, the treated piglets showed higher amounts of acetylcholine, a neurotransmitter that is linked to improved memory. Moreover, the authors noticed increases in multiple phosphatidylcholine-related metabolites in hippocampal tissue, confirming the influence of the diet. Similar results were obtained by Hellhammer and co-workers [82] in humans.

Nagai [83] studied the in vitro effects of bovine milk phospholipids on mouse neuroblastoma Neuro2a cells from endoplasmic reticulum in order to evaluate the potential of this milk fraction as a protector against neurodegenerative diseases caused by endoplasmic reticulum stress. Nagai reported that milk phospholipids can attenuate neuronal cell death because they are a source of autophagosome membrane and also activators of autophagy signaling via PKC activation. These results indicate that milk phospholipids are bioactive compounds that could diminish the risk of some neurodegenerative diseases, such as Alzheimer’s, Parkinson’s and prion diseases, according to the authors, although much follow-up research should still be done.

Schipper and co-workers [84] investigated whether cognitive development could be improved by milk lipid droplet, and they demonstrated that phospholipid-coated lipid droplets improved the cognitive function of healthy mice. Similar results were noticed by Guan and co-workers [85]; in fact, they observed that rats fed with complex milk lipid concentrate, a mixture of dairy phospholipids, showed reduced initial heading errors in a delayed probe trial and improved vascular density and neuroplasticity in the brain regions involved in memory. The same authors [86] also showed that supplementation with phospholipids improved dopamine and glutamate neurotransmission, which may be the neurobiological mechanisms. Because of that, they concluded that milk phospholipids may prevent memory decline, possibly through improving vascular and neuronal function.

The activity of phospholipids in brain health was also tested in in vivo human models. Schubert et al. [87] showed that milk phospholipids, whichincreased the availability of cortisol, may attenuate stress-induced memory impairments in a chronically-stressed man that received high PL dosage. Gurnida et al. [88] administered infant formula enriched with gangliosides (complex glycosphingolipids) to normal healthy infants; the results demonstrated beneficial effects on cognitive development in healthy infants fed with the enriched formula. This ameliorating state was attributed to the increase of the levels of serum gangliosides after supplementation.

Tanaka et al. [89] examined the effects of milk enriched with sphingomyelin on the mental, motor and behavioral development of premature infants. They found a positive correlation between SM and the development of executive function during infancy. Finally, Timby et al. [90] eradicated the gap in cognitive performance between breastfed and formula-fed infants by MFGM supplementation of infant formula that ameliorated the cognitive function.

The mechanisms underlying the associations found between PL intake and brain health may be related to several factors. An important reason could be that MFGM contains high amounts of choline derivatives (phosphocholine, glycerophosphocholine, phosphatidylcholine and sphingomyelin). The presence of these compounds is very important because choline is essential for the development of the nervous system. In fact, low choline status during pregnancy has been associated with poor cognitive development [78]. Furthermore, SM metabolites are important components of the myelin sheath that surrounds the axons. Thus, SM may promote myelination and neurotransmitter production in the brain during early infancy. Generally, it is clear from previous studies that dietary phospholipids are effective carriers of essential fatty acids, which are able to promote brain health by reducing endoplasmic reticulum-stress (this stress was associated with many neurodegenerative disorders, including Alzheimer’s disease) [79]. Moreover, thanks to their solubility in brain cell membranes, they improve the neuroplasticity of hippocampus and the striatum brain regions (improving dopamine and glutamate neurotransmission), and this is associated with better brain development and enhanced cognitive function.

Because of these benefits of PL, dietary supplementation of infant formula with dairy PL may be a useful approach to promote brain development and a good way to reduce the gap in PL intake between breastfed and infant formula-fed babies.

### 4.2. Anticancer Activity

The literature about the anticancer activity of milk phospholipids is specifically related to colon cancer [75]. Particularly, sphingolipids have been demonstrated to play significant roles in modulating colon tumor development and activation of apoptotic pathways in such cancer cells [91]. Several studies have been performed in vitro to evaluate the effects of milk phospholipids on cancer cell lines. Kuchta-Noctor et al. [92] examined the growth-modulatory effects of buttermilk phospholipids and sphingolipid fractions obtained by membrane filtration on SW480 colon cancer cells. Their results confirmed that all of the derived buttermilk fractions proved to be antiproliferative toward SW480 cells. Based on the obtained results, they recommended certain phospholipid categories, particularly SM and glycosphingolipids, as they were shown to have the strongest antiproliferative effects. Recently, Castro-Gómez et al. [93] isolated buttermilk lipid fractions with food-grade or non-food-grade solvents and evaluated their potential antiproliferative effect on nine human cancer cell lines. They demonstrated that the fractions obtained using food-grade solvents were the richest in phospho- and sphingo-lipids and showed a strongly antiproliferative activity against human ovary and colon cancer cells.

An in vivo study conducted by Snow et al. [94] on Fischer-344 rats fed with corn oil, anhydrous milk fat or MFGM demonstrated that rats fed an MFGM-rich diet had significantly fewer aberrant crypt foci than rated on the other diets, confirming that milk phospholipids may be protective against colon carcinogenesis. Maswadeh et al. [95] isolated the phospholipid fraction from camel milk and used it to encapsulate anticancer drug etoposide in liposomes. They used the liposomes to feed female BALB/c tumor-bearing mice, and their results showed that these etoposide-liposomes imparted greater survival to fibrosarcoma-bearing mice than the animals fed with free or other liposomes. Because of that, they attributed this effect to a synergistic anticancer activity between etoposide and PE from camel MFGM.

Despite the several researches about the anticancer activity of milk PL, the mechanisms behind this activity for this class of compounds is still not clear. However, when water-soluble signal messengers (that cannot traverse the lipid membrane) stimulate the cell-surface receptors, phospholipases of specific cellular PL generate different bioactive lipid mediators that have been shown to function as mediators of numerous cell responses, including apoptosis resistance. Briefly, a deficiency of PL may diminish their membrane levels and thereby promote the accumulation of ceramide and diacylglycerol that activate the caspases and induce apoptosis.

At the same time, sphingolipids’ anticancer activity is associated with their slow digestion;because of that, they remain along the entire length of both the small intestine and the colon, resulting in the presence of their metabolite ceramide and sphingosine along the intestinal tract. High levels of ceramide and sphingosine may be causing the potentially beneficial effects because they are messengers implicated in various physiological functions, like apoptosis, and targeting mitochondrial activity in colon cancer cells.

### 4.3. Metabolic Syndrome, Lipid Metabolism and Cardiovascular Diseases

Despite the high saturated fat content, no association and/or inverse relationship between the intake of milk fat containing dairy products and the risk of cardiovascular disease, coronary heart disease and stroke have been found [96]. The same authors also reported less positive results of butter compared with other dairy products on the risk of IHD mortality; this effect may be due to the lower levels of polar lipids in butter compared with other dairy products. In fact, the negative relationship between the intake of MFGM compounds and the incidence of obesity, insulin resistance, dyslipidemia and type-2 diabetes has also been described [97]. These results indicate that MFGM may be underlying the metabolic benefits of full fat dairy consumption.

Lecomte et al. [98] compared the activity of milk and soy phospholipids on postprandial lipid metabolism. Their results show that milk phospholipids were related to a faster dynamics and clearance of postprandial lipemia, which may contribute to lower long-term fasting lipemia.

Norris et al. [99] also analyzed the effects of sphingomyelin on lipid metabolism. They compared the activity of sphingomyelin from milk and egg and found that mice fed with milk SM gained significantly less weight and had reduced serum cholesterol compared to the other ones.

A study conducted by Oosting and co-workers [100] evidenced that mice fed with infant milk formula with milk phospholipids coating the fat globules showed a reduced susceptibility to obesity in adult life. In fact, mice showed a limited hypertrophic growth of white adipose tissue, probably due to the alteration of gene expression as factors regulating adipose tissue lipogenesis. Baars et al. [101,102], recently confirmed these data, indicating that phospholipids could be an important factor in the early diet with a positive impact on long-term (metabolic) health. Moreover, as reported by Kamili et al. [103], milk phospholipids decreased total liver cholesterol and triglycerides and increased fecal cholesterol excretion in mice with a high-fat diet. Their results thus suggested that milk phospholipids may be useful for hyperlipidemic patients or in those that suffer fatty liver disease, as increased phospholipid intake may increase cholesterol excretion. Similar results have also been obtained by Conway et al. [104], who demonstrated that buttermilk reduced cholesterol concentrations in men and women, primarily through inhibition of intestinal absorption of cholesterol, thereby increasing cholesterol excretion. Similar data were reported by Keller et al. [105], who evaluated the hypolipidemic and/or hypocholesterolemic effects of milk phospholipids in combination with plant sterols in healthy volunteers. They showed that plasma LDL cholesterol concentration decreased when the volunteers were supplemented by a combined high-dose of milk PL and plant sterols; instead when only high doses (6 g PL/day) of milk PL were used, LDL cholesterol increased.

Watanabe and co-workers [106] studied the effect of polar lipids separated from butter serum on plasma and hepatic cholesterol and triacylglycerol levels of obese-model mouse; they noticed a reduction of cholesterol in plasma when total polar lipid extract or an enriched ceramide fraction were supplied. Moreover, the ceramide fraction produced a significant reduction of hepatic cholesterol and triacylglycerol levels. Because of that, they hypothesized that this effect may be due to the down-regulation of stearoyl-CoA desaturase-1 by milk ceramides.

In the end, Bjørnshave and Hermansen [107] summarized the negative relationship between the consumption of milk phospholipids and the incidence of metabolic syndrome and type 2 diabetes.

The present studies suggest an effect of milk PL on downregulation of stearoyl-CoA desaturase-1 (SCD1), which leads to a decreased level of triacylglycerol and cholesterol in the liver and in the plasma. Moreover, SM has high affinity with cholesterol, which may influence its micellar solubilization.

### 4.4. Anti-Bacterial and Anti-Inflammatory Activity

Phospholipids and membrane glycoproteins contained in MFGM may affect pathogen colonization and gut barrier integrity [108]. Ten Bruggengate and co-workers [109] demonstrated that phospholipids contained in MFGM improved the in vivo resistance to diarrheagenic *Escherichia coli*. Moreover, a regular consumption of milk phospholipids was associated with a decrease of short febrile episodes in human subjects [109]. Fuller et al. [110] also confirmed the anti-infective role of milk phospholipids; in fact, they showed that a phospholipid extract of bovine MFGM was able to inhibit the infectivity properties of rotavirus.

In spite of the fact that further research is needed to corroborate the antibacterial and antivirus effects of PL, they seem to act as both inhibitors of host cell–microbe interactions (decoy effect), as well as having a direct bactericidal activity; in addition, milk PL may improve gut barrier function [108,110].

### 4.5. Skin and Hair Condition

Milk phospholipids also have beneficial effects on skin conditions. Morifuji and co-workers [111] fed female hairless mice with low and high concentration of milk phospholipids, and they reported that mice on a diet with supplemented with a high phospholipid concentration showed higher covalently-bound ω-hydroxy ceramides in the epidermis and, generally, an improved skin barrier function due to the suppression of skin inflammation. Higurashi et al. [112] carried out their experimentation on human subjects orally supplemented by a commercial SM-containing milk phospholipid concentrate containing 5.9% of SM; their results showed that subjects supplemented with phospholipids reported higher skin elasticity below the eye than control subjects. The authors attributed this beneficial effect to an enhanced synthesis of ceramides from SM that led to the production of extracellular matrix and, because of that, improved skin elasticity.

Kumura and co-workers [113] also studied the topical effects of milk phospholipids to explore the potential use of milk phospholipids for cosmetic use. They demonstrated that milk phospholipids were able to stimulate the anagen phase due to dermal penetrative components of milk PL extract that may regulate the hair cycle. These results promote the need for further research on the possible use of milk PL for the formulation of cosmetic products that may be able to stimulate hair follicle cells, allowing with this transdermal activity the regulation of the hair cycle.

## 5. Conclusions

The studies about phospholipid composition of colostrum, milk and dairy by-products have significantly increased in the last few years. The literature data show that different factors, such as mammalian genetics, lactation stages, geographical origin, diet and cultural traditions, among others, are the principal factors that influence the phospholipid content and composition. The importance of these bioactive compounds encouraged also food and analytical chemist to improve their analytical approach (from a classical to an “omics” approach) in order to obtain new information about milk and dairy phospholipids. 

Finally, the biological effects reported by these compounds show the importance of milk and dairy products as a source of phospholipids and may underlie the metabolic benefits of the consumption of full-fat dairy products. Moreover, the recovery of phospholipids from dairy by-products is interesting due to the beneficial effects that phospholipid isolates may provide to human health.

## Figures and Tables

**Table 1:** Overview of phospholipid content in colostrum, milk and dairy by-products.

Sample	Phospholipids Identified	Total PL Amounts	Year	Reference
Human colostrum and milk				
Human colostrum	PI, PS, PE, PC, SM	4.42 mg/g fat	2012	[7]
Human colostrum	PI, PS, PE, PC, SM	31.4–37.2 mg/100 mL	2016	[8]
Human milk	PI, PA	Not reported	2010	[9]
Human milk	PI, PS, PE, PC, SM	Not reported	2010	[10]
Human milk	PI, PS, PE, PC, SM	3.05–4.08 mg/g fat	2011	[11]
Human milk	LPE, EPLAS, PE, SM, PS, PI, PC	152.9–473.6 µg/mL	2012	[12]
Human milk	PI, PS, PE, PC, SM	5.06–5.86 mg/g fat	2012	[7]
Human milk	PI, PS, PE, PC, SM, LPC	182.4 mg/L	2013	[13]
Human milk	PI, PS, PE, PC, SM	12.9–38.4 mg/100 g	2013	[14]
Human milk	PI, PS, PE, PC, SM	Not reported	2015	[15]
Human milk	PI, PS, PE, PC, SM	26.0–53.5 mg/100 mL	2016	[8]
Bovine colostrum, milk and dairy by-products				
Bovine colostrum	PI, PS, PE, PC, SM	0.02–0.04 g/100 g milk	2014	[16]
Bovine colostrum	PI, PS, PE, PC, SM	4.78 mg/g fat	2015	[17]
Bovine milk	LPC, PC, SM, ePC, LPE, PE-cer, ePE, PI, PS, PA	Not reported	2010	[18]
Bovine milk	LaCcer, PE, PI, PS, PC, SM	Not reported	2010	[19]
Bovine milk	PG, PA, PI, PS, PE, PC, SM, LPC, LPE, PE-cer, ePC, ePE	Not reported	2010	[20]
Bovine milk	PI, PS, PE, PC, SM, LPC, ePC, ePE	Not reported	2010	[21]
Bovine milk	PI, PS, PE, PC, SM, LPC	46.2 µg/mL	2011	[22]
Bovine milk	PI, PS, PE, PC, SM	Not reported	2011	[23]
Bovine milk	PI, PS, PE, PC, SM	6.25 mg/g fat	2011	[3]
Bovine milk	EPLAS, PE, PS, PI, PC	63.0–483.7 µg/mL	2012	[12]
Bovine milk	PI, PS, PE, PC, SM	22.7–31.3 mg/100 mL	2013	[24]
Bovine milk	PI, PS, PE, PC, SM	4.78 mg/g fat	2013	[25]
Bovine milk	PI, PE, PC, SM	Not reported	2013	[26]
Bovine milk	PI, PS, PE, PC, SM, LPC	Not reported	2013	[27]
Bovine milk	PI, PE, PC, SM	Not reported	2013	[28]
Bovine milk	PI, PS, PE, PC, SM, LPC	195.2–413.4 mg/L	2013	[13]
Bovine milk	Lactosylated-PE, PG, PI, PE, PS	Not reported	2014	[29]
Bovine milk	PI, PS, PE, PC, SM	Not reported	2014	[30]
Bovine milk	PI, PS, PE, PC, SM	2.7–3.5 mg/g fat	2014	[31]
Bovine milk	PI, PS, PE, PC, SM	Not reported	2014	[32]
Bovine milk	PA, PI, PS, PE, PC, SM	Not reported	2014	[33]
Bovine milk	PI, PS, PE, PC, SM	Not reported	2015	[34]
Bovine milk	GluCer, LacCer, PI, PE, PS, PC, SM	0.20–0.40 mg/mL	2015	[35]
Bovine milk	PI, PS, PE, PC, SM	0.24 g/100 g fat	2015	[36]
Bovine milk	PI, PS, PE, PC, SM	Not reported	2015	[37]
Bovine milk	PI, PS, PE, PC, SM	0.81–0.90 g/100 g fat	2015	[38]
Bovine milk	PI, PS, PE, PC, SM	5.21 mg/g fat	2015	[17]
Bovine milk	PI, PS, PE, PC, SM	Not reported	2015	[15]
Bovine milk	PI, PS, PE, PC, SM	Not reported	2016	[39]
Bovine milk	PI, PS, PE, PC, SM, LPC	Not reported	2016	[40]
Bovine buttermilk	PI, PS, PE, PC, SM	Not reported	2010	[41]
Bovine buttermilk	GluCcer, LaCcer, PA, PE, PI, PS, PC, SM	Not reported	2010	[19]
Bovine buttermilk	PG, PA, PI, PS, PE, PC, SM, LPC, LPE, PE-cer, ePC, ePE	Not reported	2010	[20]
Bovine buttermilk	PI, PS, PE, PC, SM, LPC, ePC, ePE	Not reported	2010	[21]
Bovine buttermilk	LPC, PC, SM, ePC, lyso-PE, PE-cer, ePE, PI, PS, PA	Not reported	2010	[18]
Bovine buttermilk (microfiltered)	GluCer, LacCer, PI, PE, PS, PC, SM	9.95 mg/g	2011	[42]
Bovine buttermilk powder	GluCer, LacCer, PI, PE, PS, PC, SM	32.9 mg/g	2011	[42]
Bovine buttermilk	PC, PE, PI, PS, SM	80.4–124.8 g/kg of fat or 231–438 mg/kg of product	2013	[43]
Bovine buttermilk	PC, PE, PI, PS, SM	0.30–0.53 g/L	2013	[44]
Bovine buttermilk	PI, PS, PE, PC, SM, LPC, LPE	Not reported	2014	[45]
Bovine buttermilk	PI, PS, PE, PC, SM, LPC, LPE	1.44 g/kg	2015	[46]
Bovine buttermilk	PI, PS, PE, PC, SM	Not reported	2016	[39]
Bovine butter serum	PI, PS, PE, PC, SM	Not reported	2016	[39]
Bovine cream by-products	PI, PS, PE, PC, SM	3.5–3.8 mg/g fat	2015	[47]
Bovine whey	PI, PS, PE, PC, SM	31.05 g/100 g MFMG	2013	[48]
Other mammalian milk				
Buffalo milk	PI, PS, PE, PC, SM	3.22 mg/g fat	2013	[25]
Camel milk	PA, EPLAS, PE, SM, PS, PI, aaPC, PC	257.0–660.3 µg/mL	2012	[12]
Camel milk	PI, PS, PE, PC, SM	4.65 mg/g fat	2013	[25]
Donkey milk	PI, PS, PE, PC, SM	2.9 µg/mL	2011	[22]
Donkey milk	PI, PS, PE, PC, SM	4.01 mg/g fat	2013	[25]
Donkey milk	PI, PS, PE, PC, SM, LPC	32.7–38.9 mg/L	2013	[13]
Dromedary milk	PI, PE, PS, PC	60–66 µg/mL	2016	[49]
Goat milk	LaCcer, PE, PI, PS, PC, SM	Not reported	2010	[19]
Goat milk	PI, PE, PC, SM, LPC	Not reported	2013	[26]
Goat milk	PI, PS, PE, PC, SM	281.6 mg/L	2013	[50]
Goat milk	PI, PS, PE, PC, SM, LPC	195.5–202.1 mg/L	2013	[13]
Goat milk	PI, PS, PE, PC, SM	Not reported	2014	[33]
Goat milk	PI, PS, PE, PC, SM	0.05–0.08 g/g fat	2016	[51]
Mare milk	LPA, LPE, PA, EPLAS, PE, SM, PS, LPC, PI, aaPC, PC	52.6–87.9 µg/mL	2012	[12]
Sheep milk	LaCcer, PE, PI, PS, PC, SM	Not reported	2010	[19]
Sheep milk	PI, PS, PE, PC, SM	308.1 mg/L	2013	[50]
Sheep milk	PI, PS, PE, PC, SM	4.30 mg/g fat	2013	[25]
Sheep milk	PI, PE, PC, SM, LPC	Not reported	2013	[26]
Sheep milk	PI, PS, PE, PC, SM	Not reported	2014	[33]

**Table 2:** Principal extraction and analytical methods used for colostrum, milk and dairy by-products’ phospholipids’ determination.ELSD, evaporative light scattering detection.

Sample	Phospholipid Identified	Extraction Method	Determination Method	Year	Ref.
Human milk	PI, PS, PE, PC, SM	Folch method	TLC	2010	[10]
Bovine milk, bovine buttermilk	LPC, PC, SM, ePC, LPE, PE-cer, ePE, PI, PS, PA	Folch method and SPE purification	Infusion in ESI-MS/MS	2010	[18]
Bovine milk and buttermilk, goat milk, ewe milk	GluCcer, LaCcer, PE, PI, PS, PC, SM	Folch method	HPLC-ELSD	2010	[19]
Bovine milk, bovine buttermilk	PG, PA, PI, PS, PE, PC, SM, LPC, LPE, PE-cer, ePC, ePE	Folch method and SPE purification	Infusion in ESI-MS/MS	2010	[20]
Bovine milk, bovine buttermilk	PI, PS, PE, PC, SM, LPC, ePC, ePE	Folch method and SPE purification	Infusion in ESI-MS/MS	2010	[21]
Bovine buttermilk	PI, PS, PE, PC, SM	Mojonnier ether extraction method	TLC and HPLC-ELSD	2010	[41]
Human milk	PI, PA	Folch method	ESI FT-ICRMS	2010	[9]
Human milk	PI, PS, PE, PC, SM	Folch method	HPLC-ELSD	2011	[11]
Bovine milk, donkey milk	PI, PS, PE, PC, SM, LPC	Folch method and SPE purification	HPLC-ELSD HPLC-ESI-IT-TOF-MS	2011	[22]
Bovine milk	PI, PS, PE, PC, SM	Folch method	HPLC-ELSD	2011	[23]
Bovine milk	PI, PS, PE, PC, SM	Folch method	HPLC-ELSD	2011	[3]
Bovine buttermilk powder	GluCer, LacCer, PI, PE, PS, PC, SM	Folch method	HPLC-ELSD	2011	[42]
Bovine milk, Human milk, camel milk, mare milk	LPE, EPLAS, PE, PS, PI, PC, SM, LPC, LPA, aaPC	Folch method	^31^P NMR	2012	[12]
Human colostrum, human milk	PI, PS, PE, PC, SM	Folch method	HPLC-ELSD	2012	[7]
Human milk, donkey milk, bovine milk, goat milk	PI, PS, PE, PC, SM, LPC	Folch method	HPLC-ELSD HPLC-ESI-IT-TOF-MS	2013	[13]
Human milk	PI, PS, PE, PC, SM	Folch method	HPLC-ELSD	2013	[14]
Bovine milk	PI, PS, PE, PC, SM	Folch method and SPE purification	HPLC-charged aerosol detector (CAD)	2013	[24]
Bovine milk, buffalo milk, sheep milk, donkey milk, camel milk	PI, PS, PE, PC, SM	Folch method	HPLC-ELSD	2013	[25]
Bovine milk, goat milk, sheep milk	PI, PE, PC, SM, LPC	Bligh–Dyer method	MALDI-TOF-MS	2013	[26]
Bovine milk	PI, PS, PE, PC, SM, LPC	Folch method	LC × LC-MS	2013	[27]
Bovine milk	PI, PE, PC, SM	Folch method (modified) and SPE purification	HPLC-ESI-IT-MS	2013	[28]
Goat milk, ewe milk	PI, PS, PE, PC, SM	Folch and Bligh–Dyer method	HPTLC	2013	[50]
Bovine buttermilk	PC, PE, PI, PS, SM	Folch method	HPLC-ELSD HPLC-ESI-MS	2013	[43]
Bovine buttermilk	PC, PE, PI, PS, SM	Röse-Gottlieb method	^31^P NMR	2013	[44]
Bovine whey	PI, PS, PE, PC, SM	Ethanol extraction	HPTLC	2013	[48]
Bovine colostrum	PI, PS, PE, PC, SM	Folch method	HPLC-ELSD	2014	[16]
Bovine milk	PI, PS, PE, PC, SM	Folch method	HPLC-ELSD	2014	[23]
Bovine milk	Lactosylated-PE, PG, PI, PE, PS	Bligh and Dyer extraction	MALDI-TOF-MS	2014	[29]
Bovine milk	PI, PS, PE, PC, SM	Folch method	HPLC-ELSD	2014	[30]
Bovine milk	PI, PS, PE, PC, SM	Folch method	HPLC-ELSD	2014	[31]
Bovine milk, goat milk, sheep milk	PA, PI, PS, PE, PC, SM	Dichloromethane-methanol solution (2/1, *v*/*v*)/PLE	HPLC-ELSD	2014	[33]
Bovine buttermilk	PI, PS, PE, PC, SM, LPC, LPE	Bligh and Dyer method	HAP chromatography and MALDI-TOF-MS	2014	[45]
Human milk, bovine milk	PI, PS, PE, PC, SM	Chloroform/methanol 2/1 *v*/*v*	TripleTOF-MS	2015	[15]
Bovine colostrum, bovine milk	PI, PS, PE, PC, SM	Folch method	HPLC-ELSD	2015	[17]
Bovine milk	PI, PS, PE, PC, SM	Folch method	HPLC-ELSD	2015	[34]
Bovine milk	GluCer, LacCer, PI, PE, PS, PC, SM	Chloroform/methanol 2/1 *v*/*v*	HPLC-LTQ Orbitrap-MS	2015	[35]
Bovine milk	PI, PS, PE, PC, SM	Chloroform/methanol/distilled water (0.8% *w*/*v*NaCl) (8:4:3 *v*/*v*/*v*)	HPLC-ELSD	2015	[36]
Bovine milk	PI, PS, PE, PC, SM	Folch method	HPLC-ELSD	2015	[37]
Bovine milk	PI, PS, PE, PC, SM	Folch method	HPLC-ELSD	2015	[38]
Bovine buttermilk	PI, PS, PE, PC, SM, LPC, LPE	Bligh and Dyer method	HPLC-ELSD	2015	[46]
Bovine cream by-products	PI, PS, PE, PC, SM	Folch method and SPE purification	HPLC-ELSD HPLC-ESI-MS	2015	[47]
Bovine milk, bovine buttermilk, bovine butter serum	PI, PS, PE, PC, SM	Folch and Röse–Gottlieb method	HPLC-charged aerosol detector (CAD)	2016	[39]
Bovine milk	PI, PS, PE, PC, SM, LPC	Folch method and SPE purification	HPLC-MALDI-TOF/TOF-MS	2016	[40]
Dromedary milk	PI, PE, PS, PC	Folch method	HPLC-UV	2016	[49]
Goat milk	PI, PS, PE, PC, SM	Methanol-chloroform-water (1:2:0.6, *v*/*v*/*v*) and chloroform/ethanol (3%, *v*/*v*)	HPLC-ELSD	2016	[51]
Human colostrum, human milk	PI, PS, PE, PC, SM	Dichloromethane-methanol solution (2/1, *v*/*v*)	HPLC-ELSD	2016	[8]

## Abbreviations

LPC	lysophosphatidylcholine
PC	phosphatidylcholine
SM	sphingomyelin
ePC	ether phosphatidylcholine
LPE	lysophosphatidylethanolamine
PE	phosphatidylethanolamine
PE-cer	phosphoethanolamine-ceramide
ePE	ether phosphatidylethanolamine
PI	phosphatidylinositol
PS	phosphatidylserine
PA	phosphatidic acid
MFGM	milk fat globule membrane
EPLAS	phosphatidylethanolamine plasmalogen
aaPC	alkyl-acyl phosphatidylcholine
LPA	lysophosphatidic acid
LPS	lysophosphatidylserine
LaCcer	lactosylceramide
GluCcer	glucosylceramide
MFG	membrane fat globule
ARCD	age-related cognitive decline
CLA	conjugated linoleic acids
PUFAs	polyunsaturated fatty acids
SFAs	saturated fatty acids
SPE	solid phase extraction
ESI FT-ICR MS	electrospray ionization Fourier transform ion cyclotron resonance mass spectrometry
PLE	pressurized liquid extraction
HAP	hydroxylapatite chromatography
IHD	ischaemic heart disease

## References

[1] Pereira P.C. (2014). Milk nutritional composition and its role in human health. Nutrition.

[2] Parodi P.W. (2004). Milk fat in human nutrition. Aust. J. Dairy Technol..

[3] Lopez C., Briard-Bion V., Menard O., Beaucher E., Rousseau F., Fauquant J., Leconte N., Benoit R. (2011). Fat globules selected from whole milk according to their size: Different compositions and structure of the biomembrane, revealing sphingomyelin-rich domains. Food Chem..

[4] Martini M., Salari F., Altomonte I. (2016). The macrostructure of milk lipids: The fat globules. Crit. Rev. Food Sci. Nutr..

[5] Contarini G., Povolo M. (2013). Phospholipids in milk fat: Composition, biological and technological significance, and analytical strategies. Int. J. Mol. Sci..

[6] Dewettinck K., Rombaut R., Thienpont N., Le T.T., Messens K., van Camp J. (2008). Nutritional and technological aspects of milk fat globule membrane material. Int. Dairy J..

[7] Zou X.Q., Guo Z., Huang J.H., Jin Q.Z., Cheong L.Z., Wang X.G., Xu X.B. (2012). Human milk fat globules from different stages of lactation: A lipid composition analysis and microstructure characterization. J. Agric. Food Chem..

[8] Claumarchirant L., Cilla A., Matencio E., Sanchez-Siles L.M., Castro-Gomez P., Fontecha J., Alegría A., Lagarda M.J. (2016). Addition of milk fat globule membrane as an ingredient of infant formulas for resembling the polar lipids of human milk. Int. Dairy J..

[9] Argov-Argaman N., Smilowitz J.T., Bricarello D.A., Barboza M., Lerno L., Froehlich J.W., Lee H., Zivkovic A.M., Lemay D.G., Freeman S. (2010). Lactosomes: Structural and compositional classification of unique nanometer-sized protein lipid particles of human milk. J. Agric. Food Chem..

[10] Benoit B., Fauquant C., Daira P., Peretti N., Guichardant M., Michalski M.C. (2010). Phospholipid species and minor sterols in French human milks. Food Chem..

[11] Lopez C., Ménard O. (2011). Human milk fat globules: Polar lipid composition and in situ structural investigations revealing the heterogeneous distribution of proteins and the lateral segregation of sphingomyelin in the biological membrane. Colloids Surf. B.

[12] Garcia C., Lutz N.W., Confort-Gouny S., Cozzone P.J., Armand M., Bernard M. (2012). Phospholipid fingerprints of milk from different mammalians determined by 31P NMR: Towards specific interest in human health. Food Chem..

[13] Russo M., Cichello F., Ragonese C., Donato P., Cacciola F., Dugo P., Mondello L. (2013). Profiling and quantifying polar lipids in milk by hydrophilic interaction liquid chromatography coupled with evaporative light-scattering and mass spectrometry detection. Anal. Bioanal. Chem..

[14] Giuffrida F., Cruz-Hernandez C., Fluck B., Tavazzi I., Thakkar S.K., Destaillats F., Braun M. (2013). Quantification of phospholipids classes in human milk. Lipids.

[15] Sokol E., Ulven T., Færgeman N.J., Ejsing C.S. (2015). Comprehensive and quantitative profiling of lipid species in human milk, cow milk and a phospholipid-enriched milk formula by GC and MS/MS^ALL^. Eur. J. Lipid Sci. Technol..

[16] Contarini G., Povolo M., Pelizzola V., Monti L., Bruni A., Passolungo L., Abeni F., Degano L. (2014). Bovine colostrum: Changes in lipid constituents in the first 5 days after parturition. J. Dairy Sci..

[17] Zou X., Guo Z., Jin Q., Huang J., Cheong L., Xu X., Wang X. (2015). Composition and microstructure of colostrum and mature bovine milk fat globule membrane. Food Chem..

[18] Gallier S., Gragson D., Cabral C., Jimenez-Flores R., Everett D.W. (2010). Composition and fatty acid distribution of bovine milk phospholipids from processed milk products. J. Agric. Food Chem..

[19] Rodríguez-Alcalá L.M., Fontecha J. (2010). Major lipid classes separation of buttermilk, and cows, goats and ewes milk by high performance liquid chromatography with an evaporative light scattering detector focused on the phospholipid fraction. J. Chromatogr. A.

[20] Gallier S., Gragson D., Jimenez-Flores R., Everett D.W. (2010). Surface characterization of bovine milk phospholipid monolayers by langmuir isotherms and microscopic techniques. J. Agric. Food Chem..

[21] Gallier S., Gragson D., Jimenez-Flores R., Everett D. (2010). Using confocal laser scanning microscopy to probe the milk fat globule membrane and associated proteins. J. Agric. Food Chem..

[22] Donato P., Cacciola F., Cichello F., Russo M., Dugo P., Mondello L. (2011). Determination of phospholipids in milk samples by means of hydrophilic interaction liquid chromatography coupled to evaporative light scattering and mass spectrometry detection. J. Chromatogr. A.

[23] Mesilati-Stahy R., Mida K., Argov-Argaman N. (2011). Size-dependent lipid content of bovine milk fat globule and membrane phospholipids. J. Agric. Food Chem..

[24] Kiebowicz G., Micek P., Wawrzenczyk C. (2013). A new liquid chromatography method with charge aerosol detector (CAD) for the determination of phospholipid classes. Application to milk phospholipids. Talanta.

[25] Zou X., Huang J., Jin Q., Guo Z., Liu Y., Cheong L., Xu X., Wang X. (2013). Lipid composition analysis of milk fats from different mammalian species: Potential for use as human milk fat substitutes. J. Agric. Food Chem..

[26] Calvano C.D., de Ceglie C., Aresta A., Facchini L.A., Zambonin C.G. (2013). MALDI-TOF mass spectrometric determination of intact phospholipids as markers of illegal bovine milk adulteration of high-quality milk. Anal. Bioanal. Chem..

[27] Dugo P., Fawzy N., Cichello F., Cacciola F., Donato P., Mondello L. (2013). Stop-flow comprehensive two-dimensional liquid chromatography combined with mass spectrometric detection for phospholipid analysis. J. Chromatogr. A.

[28] Craige Trenerry V., Akbaridoust G., Plozza T., Rochfort S., Wales W.J., Auldist M., Ajlouni S. (2013). Ultra-high-performance liquid chromatography-ion trap mass spectrometry characterisation of milk polar lipids from dairy cows fed different diets. Food Chem..

[29] Calvano C.D., de Ceglie C., Zambonina C.G. (2014). Development of a direct in-matrix extraction (DIME) protocol for MALDI-TOF-MS detection of glycated phospholipids in heat-treated food samples. J. Mass Spectrom..

[30] Argov-Argaman N., Mesilati-Stahy R., Magen Y., Moallem U. (2014). Elevated concentrate-to-forage ratio in dairy cow rations is associated with a shift in the diameter of milk fat globules and remodeling of their membranes. J. Dairy Sci..

[31] Lopez C., Briard-Bion V., Ménard O. (2014). Polar lipids, sphingomyelin and long-chain unsaturated fatty acids from the milk fat globule membrane are increased in milks produced by cows fed fresh pasture based diet during spring. Food Res. Int..

[32] Mesilati-Stahy R., Argov-Argaman N. (2014). The relationship between size and lipid composition of the bovine milk fat globule is modulated by lactation stage. Food Chem..

[33] Castro-Gómez M.P., Rodriguez-Alcalá L.M., Calvo M.V., Romero J., Mendiola J.A., Ibañez E., Fontecha J. (2014). Total milk fat extraction and quantification of polar and neutral lipids of cow, goat, and ewe milk by using a pressurized liquid system and chromatographic techniques. J. Dairy Sci..

[34] Mesilati-Stahy R., Moallem U., Magen Y., Argov-Argaman N. (2015). Altered concentrate to forage ratio in cows ration enhanced bioproduction of specific size subpopulation of milk fat globules. Food Chem..

[35] Liu Z., Moate P., Cocks B., Rochfort S. (2015). Comprehensive polar lipid identification and quantification in milk byliquid chromatography-mass spectrometry. J. Chromatogr. B.

[36] Rodríguez-Alcalá L.M., Castro-Gómez P., Felipe X., Noriega L., Fontecha J. (2015). Effect of processing of cow milk by high pressures under conditions up to 900 MPa on the composition of neutral, polar lipids and fatty acids. LWT Food Sci. Technol..

[37] Mesilati-Stahy R., Malka H., Argov-Argaman N. (2015). Influence of glucogenic dietary supplementation and reproductive state of dairy cows on the composition of lipids in milk. Animal.

[38] Ferreiro T., Gayoso L., Rodríguez-Otero J.L. (2015). Milk phospholipids: Organic milk and milk rich in conjugated linoleic acid compared with conventional milk. J. Dairy Sci..

[39] Barry K.M., Dinan T.G., Murray B.A., Kelly P.M. (2016). Comparison of dairy phospholipid preparative extraction protocols in combination with analysis by high performance liquid chromatography coupled to a charged aerosol detector. Int. Dairy J..

[40] Walczak J., Pomastowski P., Bocian S., Buszewski B. (2016). Determination of phospholipids in milk using a new phosphodiester stationary phase by liquid chromatography-matrix assisted desorption ionization mass spectrometry. J. Chromatogr. A.

[41] Costa M.R., Elias-Argote X.E., Jiménez-Flores R., Gigante M.L. (2010). Use of ultrafiltration and supercritical fluid extraction to obtain a whey buttermilk powder enriched in milk fat globule membrane phospholipids. Int. Dairy J..

[42] Le T.T., Miocinovic J., Nguyen T.M., Rombaut R., van Camp J., Dewettinck K. (2011). Improved solvent extraction procedure and high-performance liquid chromatography evaporative light-scattering detector method for analysis of polar lipids from dairy materials. J. Agric. Food Chem..

[43] Verardo V., Gómez-Caravaca A.M., Gori A., Losi G., Caboni M.F. (2013). Bioactive lipids in the butter production chain from Parmigiano Reggiano cheese area. J. Sci. Food Agric..

[44] Konrad G., Kleinschmidt T., Lorenz C. (2013). Ultrafiltration of whey buttermilk to obtain a phospholipid concentrate. Int. Dairy J..

[45] Pinto G., Caira S., Mamone G., Ferranti P., Addeo F., Picariello G. (2014). Fractionation of complex lipid mixtures by hydroxyapatitechromatography for lipidomic purposes. J. Chromatogr. A.

[46] Svanborg S., Johansen A.G., Abrahamsen R.K., Skeie S.B. (2015). The composition and functional properties of whey protein concentrates produced from buttermilk are comparable with those of whey protein concentrates produced from skimmed milk. J. Dairy Sci..

[47] Guerra E., Verardo V., Caboni M.F. (2015). Determination of bioactive compounds in cream obtained as a by-product during cheese-making: Influence of cows’ diet on lipid quality. Int. Dairy J..

[48] Zhu D., Damodaran S. (2013). Dairy lecithin from cheese whey fat globule membrane: Its extraction, composition, oxidative stability, and emulsifying properties. J. Am. Oil Chem. Soc..

[49] Yassin A.M., Hamid M.I.A., Farid O.A., Amer H., Warda M. (2016). Dromedary milk exosomes as mammary transcriptome nano-vehicle: Their isolation, vesicular and phospholipidomic characterizations. J. Adv. Res..

[50] Zancada L., Pérez-Díez F., Sánchez-Juanes F., Alonso J.M., García-Pardo L.A., Hueso P. (2013). Phospholipid classes and fatty acid composition of ewe’s and goat’s milk. Grasas y Aceites.

[51] Argov-Argaman N., Hadaya O., Glasser T., Muklada H., Dvash L., Mesilati-Stahy R., Yan Landau S. (2016). Milk fat globule size, phospholipid contents and composition of milk from purebred and Alpine-crossbred Mid-Eastern goats under confinement or grazing condition. Int. Dairy J..

[52] Argov-Argaman N., Mida K., Cohen B.C., Visker M., Hettinga K. (2013). Milk fat content and DGAT1 genotype determine lipid composition of the milk fat globule membrane. PLoS ONE.

[53] Restuccia D., Spizzirri U.G., Puoci F., Cirillo G., Vinci G., Picci N. (2012). Determination of phospholipids in food samples. Food Rev. Int..

[54] Rombaut R., Camp J.V., Dewettinck K. (2005). Analysis of phospho- and sphingolipids in dairy products by a new HPLC method. J. Dairy Sci..

[55] Lopez C., Briard-Bion V., Menard O., Rousseau F., Pradel P., Besle J.M. (2008). Phospholipid, sphingolipid, and fatty acid compositions of the milk fat globule membrane are modified by diet. J. Agric. Food Chem..

[56] Frega N.G., Pacetti D., Boselli E., Prasain J.K. (2012). Characterization of phospholipid molecular species by means of HPLC-Tandem Mass Spectrometry. Tandem Mass Spectrometry—Applications and Principles.

[57] MacKenzie A., Vyssotski M., Nekrasov E. (2009). Quantitative analysis of dairy phospholipids by ^31^P NMR. J. Am. Oil Chem. Soc..

[58] Culeddu N., Bosco M., Toffanin R., Pollesello P. (1998). ^31^P NMR analysis of phospholipids in crude extracts from different sources: Improved efficiency of the solvent system. Magn. Reson. Chem..

[59] Gallier S., Gordon K.C., Singh H. (2012). Chemical and structural characterisation of almond oil bodies and bovine milk fat globules. Food Chem..

[60] Gallier S., Vocking K., Post J.B., van de Heijning B., Acton D., van der Beek E.M., van Baalen T. (2015). A novel infant milk formula concept: Mimicking the human milk fat globule structure. Colloids Surf. B.

[61] Nguyen H.T.H., Ong L., Beaucher E., Madec M.N., Kentish S.E., Gras S.L., Lopez C. (2015). Buffalo milk fat globules and their biological membrane: In situ structural investigations. Food Res. Int..

[62] Yao Y., Zhao G., Yan Y., Mu Y., Jin Q., Zou X., Wang X. (2016). Milk fat globules by confocal Raman microscopy: Differences in human, bovine and caprine milk. Food Res. Int..

[63] Cifelli C.J., Houchins J.A., Demmer E., Fulgoni V.L. (2016). Increasing plant based foods or dairy foods differentially affects nutrient intakes: Dietary scenarios using NHANES 2007–2010. Nutrients.

[64] Brown-Riggs C. (2016). Nutrition and health disparities: The role of dairy in improving minority health outcomes. Int. J. Environ. Res. Public Health.

[65] Wellard L., Hughes C., Watson W.L. (2016). Investigating nutrient profiling and Health Star Ratings on core dairy products in Australia. Public Health Nutr..

[66] Visioli F., Strata A. (2014). Milk, dairy products, and their functional effects in humans: A narrative review of recent evidence. Adv. Nutr..

[67] Kliem K.E., Givens D.I. (2011). Dairy products in the food chain: Their impact on health. Annu. Rev. Food Sci. Technol..

[68] Küllenberg D., Taylor L.A., Schneider M., Massing U. (2012). Health effects of dietary phospholipids. Lipids Health Dis..

[69] Park K.M., Fulgoni V.L. (2013). The association between dairy product consumption and cognitive function in the National Health and Nutrition Examination Survey. Br. J. Nutr..

[70] Sébédio J.L., Malpuech-Brugère C. (2016). Metabolic syndrome and dairy product consumption: Where do we stand?. Food Res. Int..

[71] Astrup A., Rice Bradley B.H., Brenna J.T., Delplanque B., Ferry M., Torres-Gonzalez M. (2016). Regular-fat dairy and human health: A synopsis of symposia presented in Europe and North America (2014–2015). Nutrients.

[72] Díaz-López A., Bulló M., Martínez-González M.A., Corella D., Estruch R., Fitó M., Gómez-Gracia E., Fiol M., de la Corte F.J.G., Ros E. (2016). Dairy product consumption and risk of type 2 diabetes in an elderly Spanish Mediterranean population at high cardiovascular risk. Eur. J. Nutr..

[73] Vesper H., Schmelz E.M., Nikolova-Karakashian M.N., Dillehay D.L., Lynch D.V., Merrill A.H. (1999). Sphingolipids in food and the emerging importance of sphingolipids to nutrition. J. Nutr..

[74] El-Loly M.M. (2011). Composition, properties and nutritional aspects of milk fat globule membrane—A Review. Pol. J. Food Nutr. Sci..

[75] Castro-Gómez P., Garcia-Serrano A., Visioli F., Fontecha J. (2015). Relevance of dietary glycerophospholipids and sphingolipids to human health. Prostaglandins Leukot. Essent. Fat. Acids.

[76] Haramizu S., Ota N., Otsuka A., Hashizume K., Sugita S., Hase T., Murase T., Shimotoyodome A. (2014). Dietary milk fat globule membrane improves endurance capacity in mice. Am. J. Physiol. Regul. Integr. Comp. Physiol..

[77] Haramizu S., Mori T., Yano M., Ota N., Hashizume K., Otsuka A., Hase T., Shimotoyodome A. (2014). Habitual exercise plus dietary supplementation with milk fat globule membrane improves muscle function deficits via neuromuscular development in senescence-accelerated mice. SpringerPlus.

[78] Hernell O., Timby N., Domellöf M., Lönnerdal B. (2016). Clinical benefits of milk fat globule membranes for infants and children. J. Pediatr..

[79] Conway V., Gauthier S.F., Pouliot Y. (2014). Buttermilk: Much more than a source of milk phospholipids. Anim. Front..

[80] Camfield D.A., Owen L., Scholey A.B., Pipingas A., Stough C. (2011). Dairy constituents and neurocognitive health in ageing. Br. J. Nutr..

[81] Liu H., Radlowski E.C., Conrad M.S., Li Y., Dilger R.N., Johnson R.W. (2014). Early supplementation of, phospholipids and gangliosides affects brain and cognitive development in neonatal piglets. J. Nutr..

[82] Hellhammer J., Waladkhani A.R., Hero T., Buss C. (2010). Effects of milk phospholipid on memory and psychological stress response. Br. Food J..

[83] Nagai K. (2012). Bovine milk phospholipid fraction protects Neuro2a cells from endoplasmic reticulum stress via PKC activation and autophagy. J. Biosci. Bioeng..

[84] Schipper L., van Dijk G., Broersen L.M., Loos M., Bartke N., Scheurink A.J.V., van der Beek E.M. (2016). A postnatal diet containing phospholipids, processed to yield large, phospholipid-coated lipid droplets, affects specific cognitive behaviors in healthy male mice. J. Nutr..

[85] Guan J., MacGibbon A., Zhang R., Elliffe D.M., Moon S., Liu D.X. (2015). Supplementation of complex milk lipid concentrate (CMLc) improved the memory of aged rats. Nutr. Neurosci..

[86] Guan J., MacGibbon A., Fong B., Zhang R., Liu K., Rowan A., McJarrow P. (2015). Long-term supplementation with β serum concentrate (BSC), a complex of milk lipids, during post-natal brain development improves memory in rats. Nutrients.

[87] Schubert M., Contreras C., Franz N., Hellhammer J. (2011). Milk-based phospholipids increase morning cortisol availability and improve memory in chronically stressed men. Nutr. Res..

[88] Gurnida D.A., Rowan A.M., Idjradinata P., Muchtadi D., Sekarwana N. (2012). Association of complex lipids containing gangliosides with cognitive development of6-month-old infants. Early Hum. Dev..

[89] Tanaka K., Hosozawa M., Kudo N., Yoshikawa N., Hisata K., Shoji H., Shinohara K., Shimizu T. (2013). The pilot study: Sphingomyelin-fortified milk has a positive association with the neurobehavioural development of very low birth weight infants during infancy, randomized control trial. Brain Dev..

[90] Timby N., Domellöf E., Hernell O., Lönnerdal B., Domellöf M. (2014). Neurodevelopment, nutrition, and growth until 12 mo of age in infants fed a low-energy, low-protein formula supplemented with bovine milk fat globule membranes: A randomized controlled trial. Am. J. Clin. Nutr..

[91] Kuchta A.M., Kelly P.M., Stanton C., Devery R.A. (2012). Milk fat globule membrane—A source of polar lipids for colon health? A review. Int. J. Dairy Technol..

[92] Kuchta-Noctor A.M., Murray B.A., Stanton C., Devery R., Kelly P.M. (2016). Anticancer Activity of Buttermilk Against SW480 Colon Cancer Cells is Associated with Caspase-Independent Cell Death and Attenuation of Wnt, Akt, and ERK Signaling. Nutr. Cancer.

[93] Castro-Gómez P., Rodríguez-Alcalá L.M., Monteiro K.M., Ruiz A.L.T.G., Carvalho J.E., Fontecha J. (2016). Antiproliferative activity of buttermilk lipid fractions isolated using Food grade and non-food grade solvents on human cancer cell lines. Food Chem..

[94] Snow D.R., Jimenez-Flores R., Ward R.E., Cambell J., Young M.J., Nemere I., Hintze K.J. (2010). Dietary milk fat globule membrane reduces the incidence of aberrant crypt foci in Fischer-344 rats. J. Agric. Food Chem..

[95] Maswadeh H.M., Aljarbou A.N., Alorainy M.S., Alsharidah M.S., Khan M.A. (2015). Etoposide incorporated into camel milk phospholipids liposomes shows increased activity against fibrosarcoma in a mouse model. BioMed Res. Int..

[96] Huth P.J., Park K.M. (2012). Influence of dairy product and milk fat consumption on cardiovascular disease risk: A review of the evidence. Adv. Nutr..

[97] Rueda R. (2014). The role of complex lipids in attaining metabolic health. Curr. Cardiovasc. Risk Rep..

[98] Lecomte M., Bourlieu C., Meugnier E., Penhoat A., Cheillan D., Pineau G., Loizon E., Trauchessec M., Claude M., Ménard O. (2015). Milk polar lipids affect in vitro digestive lipolysis and postprandial lipid metabolism in mice. J. Nutr..

[99] Norris G.H., Jiang C., Ryan J., Porter C.M., Blesso C.N. (2016). Milk sphingomyelin improves lipid metabolism and alters gut microbiota in high fat diet-fed mice. J. Nutr. Biochem..

[100] Oosting A., van Vlies N., Kegler D., Schipper L., Abrahamse-Berkeveld M., Ringler S., Verkade H.J., van der Beek E.M. (2014). Effect of dietary lipid structure in early postnatal life on mouse adipose tissue development and function in adulthood. Br. J. Nutr..

[101] Baars A., Oosting A., Engels E., Kegler D., Kodde A., Schipper L., Verkade H.J., van der Beek E.M. (2016). Milk fat globule membrane coating of large lipid droplets in the diet of young mice prevents body fat accumulation in adulthood. Br. J. Nutr..

[102] Oosting A., Kegler D., Wopereis H.J., Teller I.C., van de Heijning B.J.M., Verkade H.J., van der Beek E.M. (2012). Size and phospholipid coating of lipid droplets in the diet of young mice modify body fat accumulation in adulthood. Pediatr. Res..

[103] Kamili A., Wat E., Chung R.W.S., Tandy S., Weir J.M., Meikle P.J., Cohn J.S. (2010). Hepatic accumulation of intestinal cholesterol is decreased and fecal cholesterol excretion is increased in mice fed a high-fat diet supplemented with milk phospholipids. Nutr. Metab..

[104] Conway V., Couture P., Richard C., Gauthier S.F., Pouliot Y., Lamarche B. (2013). Impact of buttermilk consumption on plasma lipids and surrogate markers of cholesterol homeostasis in men and women. Nutr. Metab. Cardiovasc. Dis..

[105] Keller S., Malarski A., Reuther C., Kertscher R., Kiehntopf M., Jahreis G. (2013). Milk phospholipid and plant sterol-dependent modulation of plasma lipids in healthy volunteers. Eur. J. Nutr..

[106] Watanabe S., Takahashi T., Tanaka L., Haruta Y., Shiota M., Hosokawa M., Miyashita K. (2011). The effect of milk polar lipids separated from butter serum on the lipid levels in the liver and the plasma of obese-model mouse (KK-A^y^). J. Funct. Foods.

[107] Bjørnshave A., Hermansen K. (2014). Effects of dairy protein and fat on the metabolic syndrome and type 2 diabetes. Rev. Diabet. Stud..

[108] Ten Bruggencate S.J., Frederiksen P.D., Pedersen S.M., Floris-Vollenbroek E.G., Lucas-van de Bos E., van Hoffen E., Wejse P.L. (2016). Dietary milk-fat-globule membrane affects resistance to diarrheagenic *Escherichia coli* in healthy adults in a randomized, placebo-controlled, double-blind study. J. Nutr..

[109] Veereman-Wauters G., Staelens S., Rombaut R., Dewettinck K., Deboutte D., Brummer R.J., Boone M., Le Ruyet P. (2012). Milk fat globule membrane (INPULSE) enriched formula milk decreases febrile episodes and may improve behavioral regulation in young children. Nutrition.

[110] Fuller K.L., Kuhlenschmidt T.B., Kuhlenschmidt M.S., Jiménez-Flores R., Donovan S.M. (2013). Milk fat globule membrane isolated from buttermilk or whey cream and their lipid components inhibit infectivity of rotavirus in vitro. J. Dairy Sci..

[111] Morifuji M., Oba C., Ichikawa S., Ito K., Kawahata K., Asami Y., Ikegami S., Itoh H., Sugawara T. (2015). A novel mechanism for improvement of dry skin by dietary milk phospholipids: Effect on epidermal covalently bound ceramides and skin inflammation in hairless mice. J. Dermatol. Sci..

[112] Higurashi S., Haruta-Ono Y., Urazono H., Kobayashi T., Kadooka Y. (2015). Improvement of skin condition by oral supplementation with sphingomyelin-containing milk phospholipids in a double-blind, placebo-controlled, randomized trial. J. Dairy Sci..

[113] Kumura H., Sawada T., Oda Y., Konno M., Kobayashi K. (2012). Potential of polar lipids from bovine milk to regulate the rodent dorsal hair cycle. J. Dairy Sci..

